# Micromixer Synthesis Platform for a Tuneable Production of Magnetic Single-Core Iron Oxide Nanoparticles

**DOI:** 10.3390/nano10091845

**Published:** 2020-09-15

**Authors:** Abdulkader Baki, Norbert Löwa, Amani Remmo, Frank Wiekhorst, Regina Bleul

**Affiliations:** 1 Devision Energy and Chemical Technology, Fraunhofer Institute for Microengineering and Microsystems IMM, Carl-Zeiss-Straße 18-20, 55129 Mainz, Germany; abdulkader.baki@imm-extern.fraunhofer.de; 2 Physikalisch-Technische Bundesanstalt, 8.2 Biosignals, Abbestraße 2-12, 10587 Berlin, Germany; norbert.loewa@ptb.de (N.L.); Amani.Remmo@ptb.de (A.R.); frank.wiekhorst@ptb.de (F.W.)

**Keywords:** iron oxide magnetic nanoparticles, flow chemistry, continuous synthesis, micromixer, magnetic particle imaging, magnetic particle spectroscopy

## Abstract

Micromixer technology is a novel approach to manufacture magnetic single-core iron oxide nanoparticles that offer huge potential for biomedical applications. This platform allows a continuous, scalable, and highly controllable synthesis of magnetic nanoparticles with biocompatible educts via aqueous synthesis route. Since each biomedical application requires specific physical and chemical properties, a comprehensive understanding of the synthesis mechanisms is not only mandatory to control the size and shape of desired nanoparticle systems but, above all, to obtain the envisaged magnetic particle characteristics. The accurate process control of the micromixer technology can be maintained by adjusting two parameters: the synthesis temperature and the residence time. To this end, we performed a systematic variation of these two control parameters synthesizing magnetic nanoparticle systems, which were analyzed afterward by structural (transmission electron microscopy and differential sedimentation centrifugation) and, especially, magnetic characterization methods (magnetic particle spectroscopy and AC susceptibility). Furthermore, we investigated the reproducibility of the microtechnological nanoparticle manufacturing process compared to batch preparation. Our characterization demonstrated the high magnetic quality of single-core iron oxide nanoparticles with core diameters in the range of 20 nm to 40 nm synthesized by micromixer technology. Moreover, we demonstrated the high capability of a newly developed benchtop magnetic particle spectroscopy device that directly monitored the magnetic properties of the magnetic nanoparticles with the highest sensitivity and millisecond temporal resolution during continuous micromixer synthesis.

## 1. Introduction

Due to their unique imaging, optoelectronics, catalysis, sensing, and drug delivery properties [1,2,3], nanocarriers and nanoparticulate systems have drawn significant attention in recent decades. Especially, magnetic iron oxide nanoparticles (MNP) with their unique properties comprising high magnetic moments, good biocompatibility [4], and highly flexible surface chemistry [5], belong to a material class suitable for a wide range of biomedical applications [6,7,8]. These include cell labeling, magnetic drug targeting [9], magnetic fluid hyperthermia [10,11], and diagnostic imaging [12]. Already in 1988, MNP (Resovist^®^) had been clinically evaluated as a negative contrast agent for magnetic resonance imaging of the liver [13]. Since 2005, MNP is also in development as tracer material for magnetic particle imaging (MPI), a novel quantitative imaging technology with potential for cancer diagnosis using MNP as local probes [14].

For the versatile biomedical applications of MNP, different requirements concerning their specific structural and chemical properties have to be considered by establishing an appropriate synthesis strategy. The particle properties, including size, crystal structure, and chemical composition of the core, in combination with surface chemistry [15] and surface charge (zeta potential) [16], influence the colloidal stability in a dispersion medium [17] as well as interactions with the biological environment. However, the most significant factor contributing to the characteristics of MNP for these applications is the magnetic behavior, which is determined in addition to the chemical composition, mainly by size, size distribution, and dispersion stability [18]. Bawendi et al. showed that very small MNP with a core magnetic diameter of about 3 nm and an ultrathin hydrophilic shell of about 1 nm was superior to larger particles as a positive *T*_1_ contrast agent for magnetic resonance imaging and magnetic resonance angiography [19]. Contrary, theoretical models suggest that single-core iron oxide nanoparticles of about 30 nm core diameter are optimal for MPI [14]. The tailoring of size and morphology with an easy and controllable technique is the key to optimize the magnetic behavior of MNP for their envisaged in vivo applications. Thus, a deep and comprehensive understanding of the synthesis mechanisms is not only mandatory to control the size and shape of synthesized MNP but, above all, to obtain the envisaged magnetic particle functionality.

To date, conventional batch methods of synthesizing MNP like micro-emulsion, hydrothermal reactions, sol-gel, and coprecipitation have been mostly used [20,21,22,23,24,25,26,27,28]. The simplest route already proposed in 1980 by Khalafalalla and Reimes [29] and Massart [30] produces MNP depending on a precipitating iron salt Fe(III)/Fe(II) mixture with a base, normally NaOH, followed by oxidation. The resulting diameters in the range of 5 to 15 nm usually show a rather broad size distribution, and nanoparticles are rarely stabilized as single cores in aqueous media. An exceptional technique to produce unique MNP is biosynthesis. Faivre and Schüler used magnetotactic bacteria and magnetosomes to prepare 20–45 nm magnetite MNP with uniform morphology and excellent magnetic properties for different applications, especially for hyperthermia [31]. Probably, due to their immunogenic bacterial lipid layer containing (glyco)proteins coating, biosynthesized MNPs have not found their way into the clinic, yet. Additionally, preparation, cultivation, and harvesting of the bacteria and magnetosomes are time- and labor-intensive and take up to several weeks. Another common technique to produce MNP with narrow size distribution and good control over size and morphology is the thermal decomposition of organometallic precursor in a boiling organic solvent. Many groups use this technique to produce high-quality MNP [32,33,34]. Ferguson and Krishnan et al. presented tailored 26–28 nm ±1.5 nm single-core MNP with polyethylene glycol coating for MPI application [35,36]. Although these MNP show two to three-fold higher signal amplitudes compared to Resovist^®^, their thermal decomposition route requires heating of the mixture for at least 24 h at 320 °C, followed by a phase transfer, resulting in a rather limited product quantity. Recently, Laurent et al. reported a continuous flow approach with a simple capillary reactor via the thermal decomposition synthesis route to obtain very small iron oxide nanoparticles. In this study, different synthesis parameters were varied, such as temperatures (200 to 300 °C), pressure (5 to 100 psi), and concentration of the surfactant. The system was operated at relatively low flow rates between 0.05 and 2 mL·min^−1^, and the process included further downstream processing by multiple precipitation and resuspension steps. Variation of the synthesis parameters resulted in MNP in a relatively limited core size range between 3.4 ± 0.64 and 5.86 ± 1.38 nm [37].

Generally, conventional batch synthesis methods suffer from limited reaction control, scalability issues, and often require high temperature and post-treatment, such as washing or phase-transfer from organic solvents, leading to the time-consuming multi-step procedure. Complex methods additionally lead to pure reproducibility and cause high batch-to-batch variations of resulting particle characteristics [38]. Issues of insufficient mixing and mass and heat transfer challenges even increase with increasing batch sizes [39]. Thus, further advances in MNP development is impeded, and the transfer of academic results into the technological and medical application is often hindered. Even though during the last decade, several authors have dealt with the continuous synthesis of magnetic nanoparticles, many of these issues remain unsolved [40,41,42,43]. The described processes often do not comprise the entire production procedures, including purification and stabilization steps; thus, the single cores are not stably dispersed in an aqueous medium, but clusters are obtained. Moreover, microfluidic processes based on PDMS (polydimethylsiloxane) structures usually do not tolerate high flow rates with the consequence that they often have to be operated at very low flow rates in the microliter per minute range, which limits the throughput and scalability [44,45].

To surmount these obstacles, a microtechnological manufacturing platform using flow chemistry for the controlled synthesis of magnetic iron oxide nanoparticles via an aqueous synthesis route has been developed. Microreaction technology in flow chemistry has been known and well-studied in the last decades. Outstanding is the simplicity of the approach together with the ability to precisely control reactions’ parameters, e.g., temperature, residence time, rapid heat and mass transfer, high mixing efficacy, and hence reproducibility [46,47,48]. However, producing nanoparticles in micromixers remains challenging as precipitating solid materials can block the microchannels of the reactors and endanger the process stability. Thus, a comprehensive process understanding is required to be able to exploit the advantages of a microstructured device and successfully run a micromixer for nanoparticles’ synthesis.

In the micromixer, permanent homogenous nucleation of core seeds takes place by the reaction of two or more educts, continuously administered by pumps. Connected to the micromixer, locally separated dwell zones ensure optimal control of particle growth. Hence, the micromixer setup enables both the spatial as well as the temporal separation of nucleation and growth stages during the continuous synthesis of MNP [38].

Generally, the size and shape of MNP can be tailored by the variation of synthesis parameters, e.g., the used precursor, iron salt ratio Fe^2+^/Fe^3+^, temperature, pH, and the base used to precipitate the magnetic iron oxide core [49].

In this study, we presented the use of aqueous micromixer synthesis to produce high-quality magnetic single-core iron oxide nanoparticles using biocompatible educts. Solely by the adjustment of two significant process parameters: the temperature and the residence time during particles’ growth, a tunable production of single-core magnetic nanoparticles could be achieved. A comparison with the conventional batch approach was carried out to study the properties and reproducibility of the two methods. The influence of temperature and variation of residence time by both changing the total flow rate as well as adjusting the volume of the residence loop was demonstrated.

To determine the resulting magnetic properties of synthesized MNP, a thoroughly magnetic characterization was carried out, mainly using a technique named magnetic particle spectroscopy (MPS). MPS can be considered as 0-dimensional MPI, e.g., it detects the non-linear dynamic magnetic response of MNP exposed to an alternating magnetic field but without technical challenging gradient fields used for spatial encoding. Originally MPS was developed to assess the MPI performance of tracer materials, but here, we used MPS to determine the reproducibility of the continuous MNP manufacturing process compared to batch synthesis using the same precursors and to quantify magnetic effects in the synthesis caused by changes in reaction temperature and reaction time. Since MPS is a very fast (2 ms) and sensitive (moments below 10^−11^ Am^2^ can be detected) technique, we presented the first results using MPS directly during continuous micromixer synthesis. Furthermore, linear AC-susceptibility measurements were used to obtain supporting information about the linear magnetization dynamics and aggregation behavior of the MNP when a small oscillating magnetic field was applied. The characterization of size, size distribution, particle shape, and morphology was determined using transmission electron microscopy (TEM) and differential centrifugal sedimentation (DCS).

## 2. Materials and Methods

### 2.1. Micromixer Synthesis

Single-core iron oxide nanoparticles were synthesized by precipitation from aqueous, alkaline solutions of iron salts based on a micromixer set-up, as previously reported [50,51]. The microfluidic synthesis platform consisted of HPLC pumps (Knauer, Berlin, Germany), a caterpillar micromixer (Fraunhofer IMM, Mainz, Germany) with 10 µL inner volume to induce particle nucleation, and several temperature-controlled reaction loops (Teflon tubing) with 3.2 mm diameter at lengths in the range 5 m to 50 m to control particle growth. Educt solutions were preheated, and a thermostatic bath (Huber, Offenburg, Germany) ensured the control of the reaction temperature *T*_s_ of the micromixer as well as the residence loop (temperature stability < 1.5 K). By adjusting the length of the reaction loops and the flow rate *Q*, the residence time *t*_r_, available for particle growth, could be controlled. The synthesis platform was enhanced by a downstream-processing-module to remove reactive agents and excess of a stabilizing agent. Briefly, solutions of iron chloride, sodium nitrate as an oxidizing agent, and sodium hydroxide (all reagents were used without further purification, purity grade ≥98%, Sigma Aldrich, Schnelldorf. Germany), prepared in degassed and deionized water, were mixed in a caterpillar micromixer (Fraunhofer IMM, Mainz, Germany) with symmetric liquid ratios and piped in a temperature-controlled reaction loop. Nanoparticle dispersions were prevented from further oxidation as well as agglomeration with the addition of tannic acid (1.7 kDa, Fluka, Schwerte, Germany) as a stabilizing agent. Though tannic acid, as a natural product, is not well defined and characterized, our experiences have shown that it can be successfully utilized to enhance dispersion stability in aqueous media and is able to prevent aggregation behavior. Since tannic acid acts mainly as an electrostatic stabilizer to ensure dispersion stability also in media with higher salt concentration, further functionalization by a sterically stabilizing agent is required. This can be realized, e.g., by pegylation.

Finally, nanoparticles were purified by the removal of unreacted educts and access of a stabilizing agent via diafiltration and magnetic separation and stored at room temperature for further analysis. In the present study, the size of the magnetic nanoparticles was adjusted by the two individual process parameters: the synthesis temperature *T*_s_ and the residence time *t*_r_. While *T*_s_ was directly adjusted by the temperature chosen in the heat bath with *T*_s_ in the range of 30 to 65 °C, there were two ways to set the residence time *t*_r_, either by the tubing length *L* of the dwell zone or by flow rate *Q* in the range of 0.6 to 11.3 min. To avoid any residence time distribution deterioration generally observed in long tubing, the inner diameter of the tubes should not be larger than 4 mm for the flow rates used in our experiments. At larger diameters, we observed a significant increase in heterogeneity of the resulting particles. Other parameters, including educts solutions, mixing ratios, and flow rates, were kept constant. With our laboratory setup, a temperature and reaction time-dependent production yield in the range of 20–50% could be achieved. The yield was calculated from total iron content used in the starting reaction mixture relative to the amount quantified by phenanthroline protocol (described below in the section: photospectroscopical determination of iron concentration) of the final product after purification. We observed the general trend that with increasing temperature and residence time, the production yield increased, e.g., at *T*_s_ = 52.5 °C from about 21% at *t*_r_ = 3.4 min to 48% at *t*_r_ = 7.5 min).

Within 3 h, a total volume of more than 4 L could be provided with our current lab-scale set-up. By adapting the inner dimensions of the microfluidic mixer (internal scale-up) or by using several mixers in parallel (parallelization scale-up), the throughput can be increased easily by a factor of 15 [52,53].

Additionally, MNPs were synthesized by a batch precipitation approach in a flask (total volume 100 mL) with preheated educt solutions, vigorously mixed, and stirred using the same reagents and reaction times to determine batch-to-batch variations compared to micromixer MNP.

### 2.2. Physicochemical Characterization

#### 2.2.1. Transmission Electron Microscopy (TEM)

TEM measurements were performed with a Zeiss Libra 120 (Zeiss, Oberkochen, Germany) electron microscope of nanoparticles’ dispersion dried on carbon-coated copper grids at 120 kV acceleration voltage. A magnetic field was applied to the grids for a short period of time (about 10 min) to accumulate MNP for subsequent TEM imaging. The images were taken by a CCD camera, and from a selection of *N* > 5000 individual nanoparticles, the mean diameter and standard deviation of the core diameter were determined automatically using the open-source software ImageJ (National Institutes of Health, Bethesda, MD, USA).

#### 2.2.2. Differential Centrifugal Sedimentation (DCS)

Accurate information on the dispersion properties of MNP in colloidal systems can be obtained by DCS (often termed analytical ultracentrifugation), where the measured sedimentation properties of the particle are directly linked to the hydrodynamic particle size distribution [54].

In contrast to characterization techniques based on light scattering, the measurement of even broad size distributions or highly light-absorbing materials is possible by DCS as different size fractions become differentially accelerated in the gravitational field. Compared to TEM, the statistics obtained in this ensemble method were much better due to the fact that the counting of individual particles is not necessary. DCS measurements were performed at 20,000 rpm (=21,504 relative centrifugal force) (CPS Instruments Inc. Measurements, Darmstadt, Germany) after calibration with a silicon dioxide (SiO_2_) standard (255 nm). The sucrose gradient was built up using 24% to 8% sucrose. Peak maximum and full width at half maximum (FWHM) were evaluated using Origin^®^ software (ADDITIVE Soft- und Hardware für Technik und Wissenschaft GmbH, Friedrichsdorf, Germany).

#### 2.2.3. Photospectroscopical Determination of Iron Concentration *c*(Fe)

The iron concentration *c*(Fe) of the nanoparticle samples was determined photospectroscopically following a phenanthroline protocol [55]. Ten microliters of nanoparticles were dissolved in 20 µL hydrochloric acid (37%). After complete dissolution, 470 µL of H_2_O was added. Fifty microliters of hydroxylamine hydrochloride (10%) and 150 µL of 1,10-phenanthrolinehydrochloride (0.1%) were added to the 50 µL of this solution. After a reaction time of 15 min, the absorbance of the formed ferroin complexes was measured photospectroscopically by a microplate reader at a wavelength of 510 nm (SpectraMax Plus 348, Molecular Devices, CA, USA), and the iron concentrations were calculated using an iron standard calibration curve (ICP (inductively coupled plasma) iron standard, *c*(Fe) = 1.25 µg/mL to 80 µg/mL, Merck, Darmstadt, Germany).

### 2.3. Magnetic Characterization

#### 2.3.1. Magnetic Particle Spectroscopy (MPS)

MPS measurements of single nanoparticle samples were performed using a commercial magnetic particle spectrometer (MPS-3, Bruker Biospin, Etllingen, Germany) operating at an amplitude *B*_ex_ = 25 mT and a frequency *f*_0_ = 25 kHz. MPS detected the non-linear dynamic magnetic response of MNP exposed to an alternating magnetic field, from which their MPI performance could be assessed. For the measurement, a fast reaction tube (Applied Biosystems^®^, MicroAmp, ThermoFischer, Schwerte, Germany) containing a sample volume of 30 µL was placed in the detection coil of the MPS system. The induced magnetization could be measured simultaneously by the coils. By Fourier transform of the detected time signal, the spectral components of an MPS measurement were obtained, showing distinctive amplitudes at odd multiples (harmonics) of the excitation frequency *f*_0_. We used three characteristic parameters of the MPS harmonic spectra, the amplitude of the third harmonic normalized to the iron amount of the sample, *A*_3_*, the (concentration-independent) ratio between 5th and 3rd harmonic, *A*_5_/*A*_3_, and the phase of the 3rd harmonic *ϕ*_3_. All values were correlated to the MPI performance with the general observation that the higher the *A*_3_* and *A*_5_/*A*_3_, the better the MPI images. The phase *ϕ*_3_ described how well the particle moments could follow the excitation field at the given frequency *f*_0_. Generally, it is observed that the smaller the particle, the better the magnetic moments follow the excitation field, and the closer is the value of *ϕ*_3_ to zero. For comparison, we used measurements of the MRI liver contrast agent Resovist with an *A*_3_* = 8.7 Am^2^/kg(Fe) at 25 mT [56] excitation amplitude. Resovist is appreciated as a gold standard due to good dynamic magnetic properties in MPS. Though withdrawn from the market, the precursor Ferucarbotran, offering the same magnetic properties, can be purchased from Meito Sanyo, Japan.

Online-MPS measurements were performed using a novel benchtop magnetic particle spectrometer with an integrated flow cell so that it could be connected directly to the growth stage of the micromixer platform. This device was recently developed at Physikalisch-Technische Bundesanstalt and is described in detail in [57]. It operates at the same basic frequency of 25 kHz as the commercial MPS device and is calibrated using reference samples measured with the calibrated commercial MPS device before. All online-MPS measurements were carried out at *B*_e_ = 12 mT to avoid any heat transfer contribution from the heating of the excitation coil of the device that might influence the synthesis of nanoparticles.

#### 2.3.2. Alternating Current Susceptibility (ACS)

**Figure A1:**
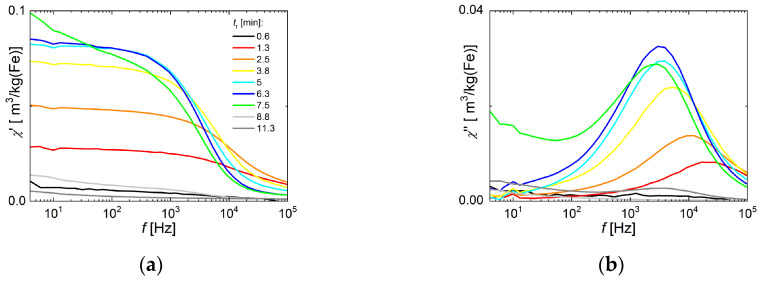
AC susceptibility versus frequency of continuous micromixer samples synthesized at different residence times adjusted by tubing length. (**a**) real part *c*′, (**b**) imaginary part *c*″.

**Figure A2:**
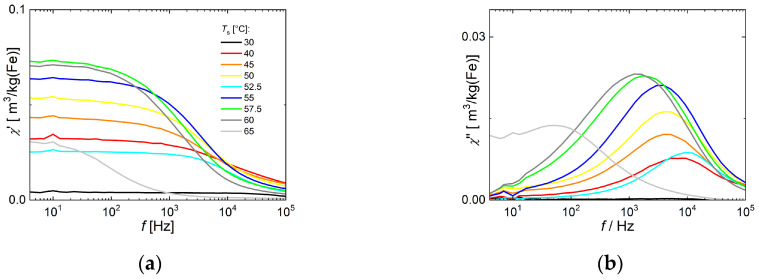
AC susceptibility versus frequency of continuous micromixer samples synthesized at different reaction temperatures *T*_s_. (**a**) real part *c*′, (**b**) imaginary part *c*″.

The linear dynamic magnetic susceptibility of MNP at room temperature was determined using an AC susceptometer (DynoMag, RISE Acreo, Goeteborg, Sweden). The complex linear dynamic magnetization response *χ*′, *χ*″ of 100 µL stock MNP suspension filled into a glass vial was measured in the frequency range 1 Hz to 100 kHz at an excitation amplitude of 0.2 mT. Typical ACS spectra normalized to the iron amount are shown in the appendix for single-core iron oxide nanoparticles synthesized at different residence times *t*_r_ (Figure A1) and as a function of synthesis temperature *T*_s_ (Figure A2). From the measurements, the initial mass susceptibility *χ*_ini_ (*χ*′ normalized to the sample’s iron mass and extrapolated to *f* → 0 Hz) in units of m^3^/kg(Fe) and the frequency *f*_p_= *f*(max(*χ*″(*ω*))) were extracted. We used *χ*_ini_ to assess the particle moment (*χ*_ini_ is proportional to the square of the particle moment, and *f*_p_ is a magnetic measure of the hydrodynamic diameter). The overall shape of the spectra was used as an indicator if aggregation occurred in a sample.

#### 2.3.3. X-ray Diffraction (XRD)

X-ray diffraction scans at room temperature were carried using a diffractometer (Rigaku SmartLab diffractometer, Neu-Isenburg, Germany) in parallel beam reflection geometry, equipped with a monochromatic copper radiation source (λ = 1.540593 Å). The patterns were collected in the 20–70° (2θ) range with a scan step of 0.01° at a speed of 0.1°/min. The main peak positions of the XRD spectra were analyzed according to the *JCPDS* card no. #*75*-0033 (Fe_3_O_4_) and # 39-1346 (*γ*-Fe_2_O_3_).

## 3. Results and Discussion

**Figure A3:**
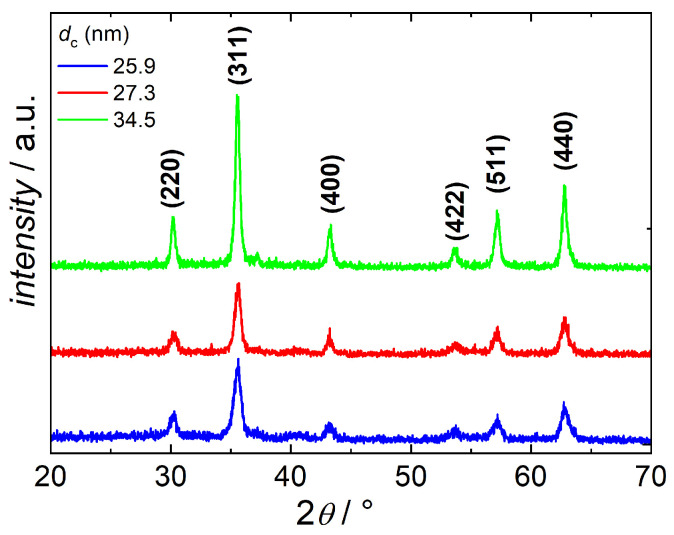
XRD spectra of three micromixer samples with average core diameters *d*_c_ of about 25 nm (blue), 27 nm (red), and 35 nm (green) obtained by micromixer synthesis at different residence times *t*_r_ at a synthesis temperature of *T*_s_ = 52 °C.

Magnetic single-core iron oxide nanoparticles were manufactured using a micromixer synthesis platform via an aqueous synthesis route. Additionally, batch syntheses employing identical educt solutions were carried out to assess performance and reproducibility between continuous micromixer and batch synthesis approach. For a comprehensive understanding of the particle formation process during continuous synthesis, the influence of the two main parameters—reaction temperature *T*_s_ and reaction time *t*_r_—on particle properties were investigated. To evaluate the magnetic properties of the single-core nanoparticles, MPS and ACS measurements were carried out. In addition to the magnetic measurements, XRD measurements of three samples with core sizes in the range 25 nm to 35 nm were performed to confirm the crystal structure and iron oxide phase (Appendix A Figure A3). Furthermore, the physicochemical analysis was augmented by TEM to assess core size and morphology and DSC to determine the hydrodynamic diameter size distribution. Since MPS is an outstanding sensitive (magnetic moments below 10^−11^ Am^2^ can be detected) and fast (2 ms for a single measurement) technique, its capability for magnetic online analysis during the continuous MNP production process was presented for the first time. Thus, direct quantitative monitoring of relevant process parameters during synthesis becomes feasible.

### 3.1. Reproducibility of Continuous Micromixer Synthesis Compared to Conventional Batch Synthesis

The magnetic properties of iron oxide nanoparticles depend on their composition, crystal structure, crystal quality, and morphology. Hence, a suitable synthesis route should be selected, which allows optimum control over shape, size, size distribution, and crystallinity of the particles. Several different synthesis routes to produce iron oxide nanoparticles exist. Well-established methods are thermal decomposition, sol-gel-approaches, mini emulsion, as well as coprecipitation [58]. Though all batch methods are based on different synthetic routes, each of them is usually running in a reaction vessel or flask at a predetermined reaction temperature while stirring. Thus, inhomogeneities in mixing and temperature within the reaction volume cause concentration gradients and hot spots, which influence the product quality. With increasing reaction volume, sufficient mixing and adequate reaction temperature control become even more challenging. For these reasons, the continuous synthesis approach of using microfluidics has gained a strong interest in recent years. However, particularly the synthesis of inorganic nanoparticles remains challenging because precipitated solids tend to block the microchannels and endanger the process stability.

**Figure 1:**
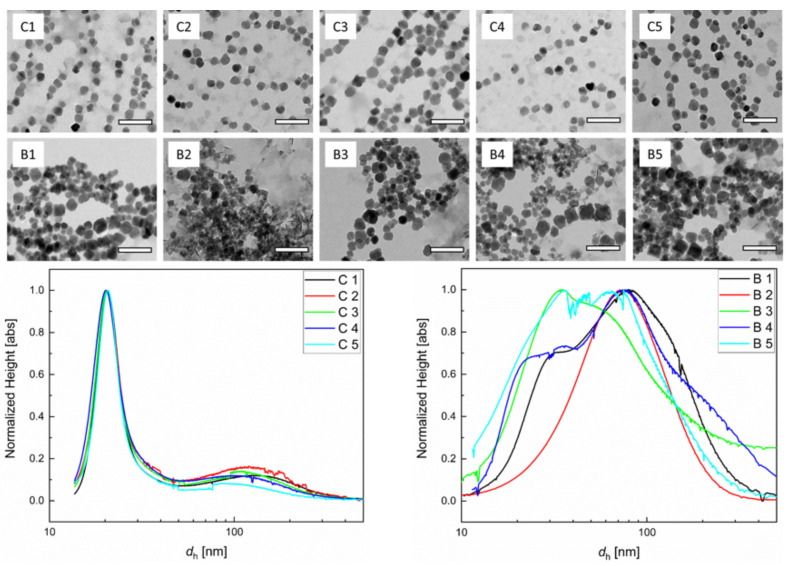
Comparison of structural nanoparticle properties between continuous micromixer synthesis and batch synthesis: Representative transmission electron microscopy (TEM) images of five individual continuous micromixers (C1–C5) at a total flow rate of 8 mL/min and batch (B1–B5) synthesis runs with 100 mL total reaction volume (scale bar 100 nm). Experiments were performed with preheated educt solutions at 52.5 °C, mixing ratio of 1:1, and a reaction time of 2.5 min. Bottom: Corresponding hydrodynamic diameter *d*_h_ distribution determined by differential centrifugal sedimentation (DCS) measurements of these samples (left: continuous micromixer synthesis, right: batch synthesis). Each line depicts the size distribution of one individual synthesis run. The narrower size distribution and higher homogeneity of the magnetic iron oxide nanoparticles (MNP) synthesized by continuous micromixer synthesis approach compared to batch synthesis were clearly visible in both TEM and ACS measurements.

We investigated the process reproducibility and stability of MNP obtained from our micromixer approach with particles manufactured by a conventional batch coprecipitation. We chose one specific set of synthesis parameters where we expected to obtain single-core nanoparticles with core sizes below 25 nm. In this case, a stable aqueous dispersion was easily achieved since interparticle interactions at these small diameters were rather negligible. Since we intended to show how insufficient mixing efficiency leads to inhomogeneous seed formation and, consequently, to broader size distribution, we did not investigate the influence of the different synthesis parameters here. To this end, we performed five individual continuous synthesis runs and compared the resulting nanoparticle samples with the properties of samples synthesized by five analogous batch synthesis runs using identical parameters. For the batch synthesis, we prepared 100 mL total volume with preheated educt solutions in a temperature-controlled reaction vial. Analogous to the continuous synthesis run, it was mixed vigorously in a 1:1 ratio at 52.5 °C and stirred for a reaction time of 2.5 min. The physicochemical characterization of representative nanoparticle samples by TEM and DSC for both synthesis routes is displayed in Figure 1.

The TEM images clearly indicated that core size distribution and morphology for the continuously manufactured samples were superior over the batch synthesis samples. Since the nucleation that determines the size distribution is a very fast process, the efficient mixing in the micromixer synthesis ensures a more homogenous distribution of core seeds, which, in the second stage, can uniformly grow at similar velocities because of the homogenous distribution of ripening educts as well as homogenous temperature conditions. This was confirmed by DSC measurements, which showed a larger variation of the hydrodynamic size distribution within the individual synthesis runs for the batch samples. Furthermore, the size distribution of the batch samples was generally broader and showed aggregates with average sizes even above 100 nm, while the core diameters were less than 30 nm. The DSC measurements of the continuous micromixer samples displayed similar and narrow hydrodynamic size distributions with relative standard deviations σ < 0.2. We attributed the observed stronger variation in hydrodynamic diameters in the batch synthesis to be caused by the more inhomogeneous seed nucleation conditions due to incomplete or insufficient mixing of educts compared to the micromixer approach. This resulted not only in a broader distribution of core sizes but in the cohesion of cores, as well. As a consequence, this led to observed increased hydrodynamic diameters.

**Figure 2:**
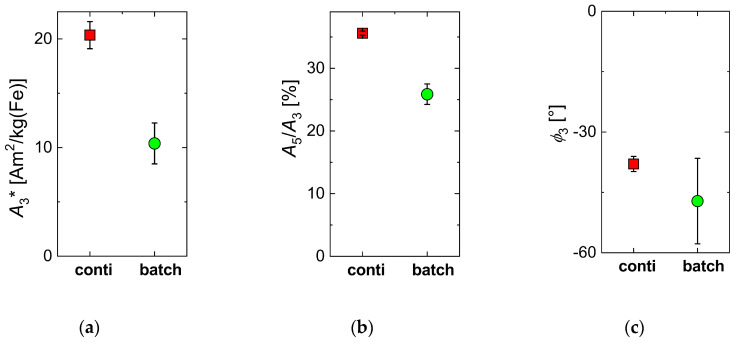
Mean (symbols) and standard deviation (displayed as uncertainty bars) of the variation of magnetic particle spectroscopy (MPS) parameters (*B*_e_ = 25 mT) of MNP samples synthesized by the two different synthesis approaches, micromixer (squares) and conventional batch (circles) procedure. For each synthesis procedure, five individual runs were carried out. (**a**) specific amplitude *A*_3_*; (**b**) ratio *A*_5_/*A*_3_; (**c**) phase *ϕ*_3_.

Regarding the magnetic properties of the five individual synthesis runs, Figure 2 shows the mean and the standard variation (plotted as uncertainty bar) of the characteristic MPS parameters *A*_3_*, *A*_5_/*A*_3_, and *ϕ*_3_ of the five individual runs synthesized either by micromixer (red) or batch synthesis (green). The specific amplitude *A*_3_* was a measure of the sensitivity of the MNP to give a dynamic magnetization response at a chosen excitation frequency *f*_0_ per unit amount of iron, whereas the ratio *A*_5_/*A*_3_ could be considered as a parameter to describe the shape of the harmonics spectrum [59,60,61]. Finally, phase *ϕ*_3_ was related to the relaxation effects of the MNP and parametrized the lag of the MNP moments to follow the excitation field. Though the same general synthetic route and the same chemical reagents were used, the MNP resulting from the micromixer synthesis showed a higher MPS performance and a much smaller variation of *A*_3_*, indicating the superior defined process control. The resulting value for *A*_3_* = 20(1) Am^2^/kg(Fe) of the micromixer samples was about twice the value of *A*_3_* = 10(2) Am^2^/kg(Fe) found for the batch samples, while the standard deviation of the batch samples was twice the value of the micromixer samples. Note, the value in brackets denotes the format standard variation of the five samples, e.g., 20(1) Am^2^/kg(Fe) = 20 +/− 1 Am^2^/kg(Fe). Both samples exhibited a higher specific MPS amplitude compared with *A*_3_* = 8.7 Am^2^/kg(Fe) reported for Resovist^®^ at 25 mT excitation amplitude [56]. Showing such high signal amplitudes, MNPs obtained by micromixer synthesis MNP were capable of competing with single-core iron oxide nanoparticles synthesized by sophisticated thermal decomposition synthesis with subsequent phase transfer [36]. Similarly, the mean value of *A*_5_/*A*_3_ = 35.6(3)% of the micromixer samples was far above the value of *A*_5_/*A*_3_ = 26(2)%, with six times higher standard deviation of the batch synthesis samples. This was a consequence of the much broader and more varying core and hydrodynamic size distributions found by TEM and DCS for the batch synthesis samples, which led to reduced *A*_3_ and *A*_5_/*A*_3_ values. Accordingly, the mean value and standard deviation of phase *ϕ*_3_ were increased (note the negative sign to emphasize the increased phase lag) for the batch synthesis samples.

In a simplified description, the non-linear dynamic response, on which MPS relies, crucially depends on the size and mobility of the magnetic moments of the nanoparticles, which are exposed to the magnetic excitation field. The larger the magnetic moment of the particle, the larger the values of *A*_3_* and *A*_5_/*A*_3_ will be, as long as the moments can follow the excitation. With the increasing magnetic moment, its mobility to align (or switch) with the excitation field will decrease, which, in turn, will lead to a phase lag, e.g., increasing (by convention negative) *ϕ*_3_ value and resulting decrease of the two parameters *A*_3_* and *A*_5_/*A*_3_. Since the size of the magnetic moment is mainly determined by the particle size and core crystal quality, whereas the mobility is given by the viscosity of the suspending medium and by the effective anisotropy, there will be an optimum particle size at a given MPS excitation frequency *f*_0_. Smaller particles (low moments with high mobility) will have reduced values of *A*_3_* and *A*_5_/*A*_3_, and a *ϕ*_3_ value close to zero, while particles larger than the optimum size (huge moments but with low mobility) will also show reduced values of *A*_3_* and *A*_5_/*A*_3_ and an increased *ϕ*_3_ value—above the optimum phase value. Therefore, samples with broad distributions will always exhibit reduced MPS parameters compared to a sample with optimum particle size and crystal structure for a given frequency *f*_0_.

### 3.2. Influence of Residence Time Adjusted by the Total Flow Rate

The micromixer synthesis platform provided the spatial and temporal separation of nucleation and growth. Core seeds formed in the micromixer at the first contact of the educts were then piped into the residence loop where subsequent particle ripening took place, e.g., the cores grew to a certain size depending on the time as well as the temperature in the residence loop. Different effects influencing the MNP properties could become addressable by changing the mixing profile (or efficiency) and the duration the particles stay in the growth stage. The residence time *t*_r_ was one main parameter that could be used to accurately control the MNP growth in continuous micromixer synthesis. With increasing *t*_r_, larger MNPs were obtained because of the longer time interval, and the seed particles produced in the micromixer resided in the growth stage. One possibility to adjust the residence time was to change the total flow rate. The higher the flow rate, the shorter the residence time in the growth stage (assuming a constant loop volume); thus, particles had less time to grow, leading to smaller particles. Reversely, lower flow rates implied longer residence times, resulting in larger particles.

However, the total flow rate not only determined the residence time but, at the same time, affected the mixing efficiency in the micromixer stage. Optimal mixing could be achieved in a relatively small window only. Since the micromixer is a static mixer without rotation or stirring elements, its mixing efficiency strongly depends on the flow conditions [62].

Therefore, the influence of the residence time *t*_r_ adjusted by the total flow rate on the structural and magnetic nanoparticle characteristics was investigated. To this end, total flow rates were varied from 4 mL/min to 18 mL/min, which corresponded to residence times from *t*_r_ = 7.5 min to 1.7 min for a constant residence volume *V* = 30 mL, since *t*_r_ = *V*/*Q*.

**Figure 3:**
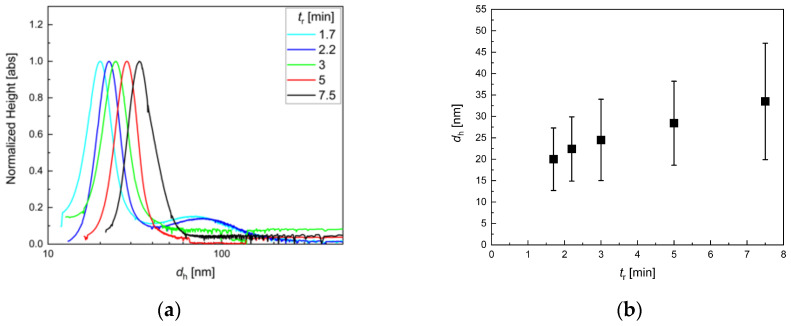
DCS analysis for iron oxide single-core nanoparticles manufactured by continuous micromixer synthesis at different total flow rates *Q* from 4 to 18 mL/min (resulting residence times *t*_r_ depicted in the legend). The reaction temperature was kept at 52.5 °C. (**a**) Hydrodynamic particle size distributions as measured by DCS; (**b**) Resulting peak maximum (symbols), resembling the mean diameter, and full width at half maximum (FWHM) (plotted as uncertainty bars), indicating the width of size distribution. Varying the total flow rate was one possibility to manipulate the particle size in the growth stage. The higher the flow rate, the smaller the resulting particles since the time for particle growth was too short for reaching an equilibrium in the residence loop.

As expected, the average particle size decreased with increasing flow rate since residence time *t*_r_ was decreased. In the parameter window investigated, mean hydrodynamic MNP diameters ranging from about 20 nm to 33 nm (Figure 3) were found. Larger particles displayed a slightly more broadened size distribution (displayed as uncertainty bars in Figure 3b); nevertheless, no larger agglomerates with diameters larger than 50 nm were detected. Thus, the chosen flow rate range of 4 to 18 mL/min resembled a reliable process parameter window in the present setup.

**Figure 4:**
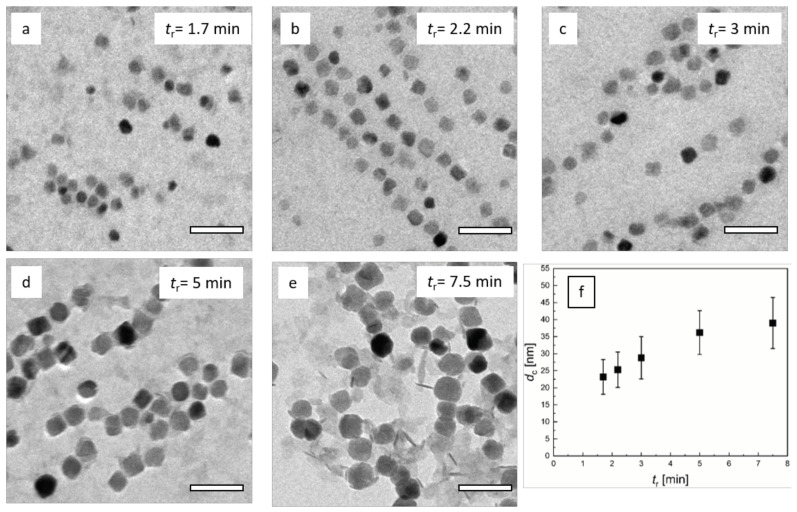
TEM analysis of micromixer nanoparticle synthesis at a reaction temperature of 52.5 °C. **a**–**e**: Variation of residence time *t*_r_ by adjusting the flow rate. Residence times from 1.7 min (*Q* = 18 mL/min) to 7.5 min (*Q* = 4 mL/min). With increasing *t*_r_, particle size increased, and homogeneity slightly decreased for the largest particles. **f**: Resulting mean diameter (symbol) and standard deviation (uncertainty bar) determined by analysis of *N* > 5000 individual nanoparticles imaged by TEM as a function of *t*_r_.

TEM data (Figure 4) confirmed the DCS observations. The core sizes increased in the same way as the hydrodynamic sizes. Moreover, particles, particularly at lower *t*_r_, were clearly separated from each other again, confirming the stable dispersion as single cores in aqueous media. This was rarely observed for particles from aqueous synthesis, even for very small cores obtained from continuous synthesis with the aid of a T-mixer [63]. However, at lower flow rates, the particle homogeneity was slightly lower than at higher flow rates. This indicated that the micromixer was not operated at optimum flow conditions, and thus, the mixing efficiency was not sufficient to ensure a homogenous distribution of core seeds, leading to the broader size distribution observed for the final particles. Furthermore, at very high flow rates (=lower *t*_r_), the final concentration seemed to decrease, indicating that, in this case, not only the core size but also the total number of seeds was reduced or that the reduced residence time allowed not all seeds to form magnetic cores.

**Figure 5:**
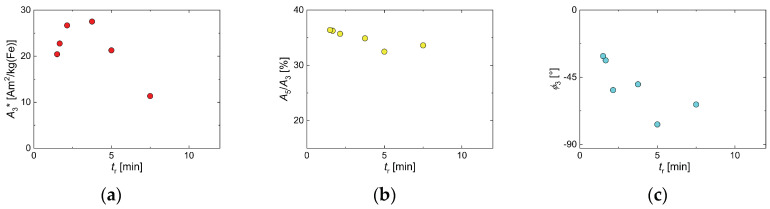
MPS parameters of MNP samples obtained by continuous micromixer synthesis at different residence times *t*_r_ = 1.7, 2.2, 3, 5, 7.5 min by adjusting the flow rate *Q*. (**a**) specific amplitude *A*_3_*; (**b**) ratio *A*_5_/*A*_3_; (**c**) phase *ϕ*_3_.

The impact of the residence time adjusted by the flow rate on the dynamic magnetic properties of the MNP, as characterized by the three MPS parameters *A*_3_*, *A*_5_/*A*_3_, and *ϕ*_3_, is shown in Figure 5. While the specific amplitude exhibited a distinct maximum of *A*_3_* ≈ 28 Am^2^/kg(Fe) at a residence time *t*_r_ of about 3 min, the ratio *A*_5_/*A*_3_ showed a weak decrease with increasing *t*_r_, only. The phase *ϕ*_3_ showed the same, but more pronounced trend as *A*_5_/*A*_3_. Since at diameters *d*_c_ > 20 nm, the magnetic moments of the MNP were blocked at room temperature, we assumed that the dynamics of the MNP moments were determined by Brownian rotation (the whole particle was rotating to follow the MPS excitation field). Therefore, we found, at the excitation parameters used for MPS (*f*_0_ = 25 kHz, *B*_e_ = 25 mT), a corresponding core size of about 25 nm (see Figure 4f), and hydrodynamic diameter of about 27 nm (Figure 3b).

The ACS measurements (Appendix A Figure A1) confirmed that with increasing *t*_r_, the resulting initial susceptibility *χ*_ini_ (e.g., core size) was increasing. Accordingly, the peak frequency *f*(*χ*″), which was related to the hydrodynamic diameter of the MNP, was shifting towards lower frequency with increasing *t*_r_, e.g., the resulting particle became larger, slowing down the Brownian rotation. A slight aggregation tendency of larger MNP produced at lower flow rates resulting in residence times ≥ 5 min *Q* < 20 mL/min was observed in the ACS measurements indicated by distorted, broader spectra with a pronounced shift of *f*(*χ*″) towards lower frequencies (supplemental data shown in Appendix A). The tendency of agglomeration was observed also for larger single-core nanoparticles with core sizes of 27.8 nm, which are synthesized by thermal decomposition during the phase transfer [16].

### 3.3. Influence of Residence Time Adjusted by the Volume of the Growth Stage Tubing

An alternative way to adjust the residence time *t*_r_ was by selecting the reaction volume of the growth stage, e.g., by the length of the tubing of the growth section. In this case, *t*_r_ could be adjusted while keeping all other synthesis parameters constant, e.g., without any further secondary effects as inhomogeneities caused by insufficient mixing (e.g., at very low flow rates). To this end, we varied, at *Q* = 8 mL/min total flow rate and *T*_s_ = 52.5 °C, the length of the tubing to adjust *t*_r_, while the inner diameter was kept constant to avoid flow profile changes by modifying the tubing diameter. By this, *t*_r_ in the range 1 min to 12 min (corresponding to a tubing volume of 5 mL to 90 mL) was adjusted, which was slightly larger than the range that was addressed by the flow rate, as presented in Section 3.2.

**Figure 6:**
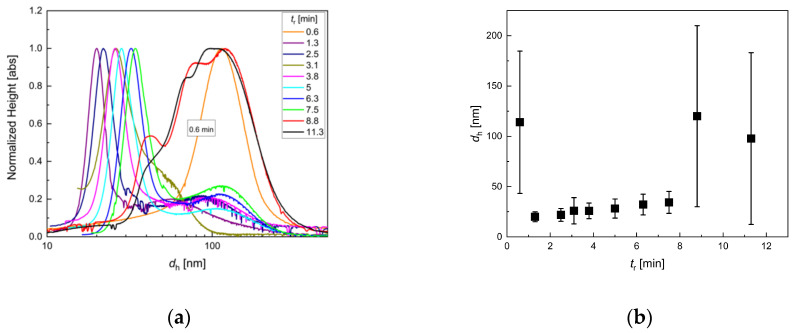
DCS analysis for iron oxide single-core nanoparticles manufactured by continuous micromixer synthesis at *Q* = 8 mL/min total flow rate and *T*_s_ = 52.5 °C for various residences times *t*_r_ = 0.6, 1.3, 2.5, 3.1, 3.8, 5, 6.3, 7.5, 8.8, 11.3 min: (**a**) hydrodynamic size distribution extracted from DCS measurements; (**b**) mean hydrodynamic diameter *d*_h_ (symbols) and FWHM values (plotted as uncertainty bars) as a function of *t*_r_.

The DCS analysis for samples synthesized at different residence times *t*_r_ identified an optimal process window, wherein a safe control over particles’ characteristics was feasible (Figure 6a). In the *t*_r_ range from 1.3 min to 7.5 min, DCS revealed narrow monomodal hydrodynamic size distributions with mean diameters of about 18 nm to 25 nm. Analogous to the experiments with a variation of the total flow rate, again, the average particle size in this process parameter range increased gradually with increasing *t*_r_ For longer residence times *t*_r_ = 8.8 min and 11.3 min, multimodal and broad size distributions became visible in the DCS analysis, as visualized by the uncertainty bars in Figure 6b. This clearly indicated agglomeration or aggregation behavior due to larger magnetic moments of larger cores. At a certain critical core size, the stabilizing agent could no longer protect the strong particle-particle interactions, and agglomeration occurred by forming superstructures. This was partly reversible, and the superstructures could be dissembled by stirring or vortexing; nevertheless, also stable aggregates were formed that could not be separated anymore.

**Figure 7:**
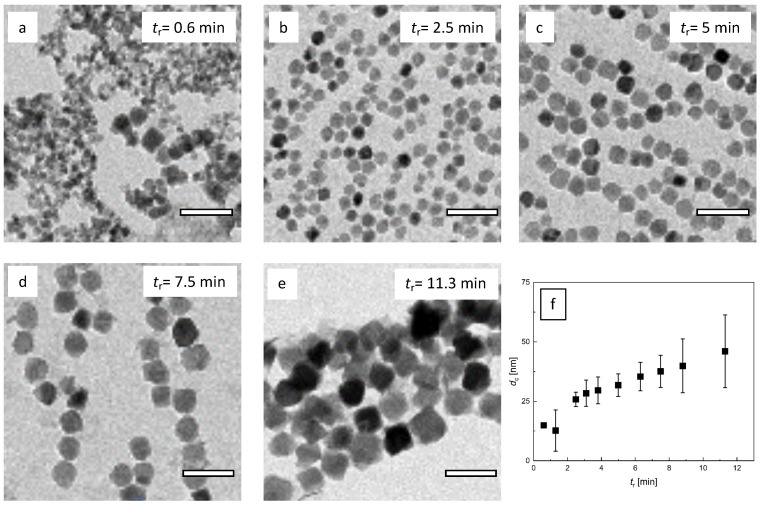
TEM analysis of micromixer nanoparticle synthesis at *Q* = 8 mL/min total flow rate and *T*_s_ = 52.5 °C. **a**–**e**: Variation of residence time *t*_r_ by adjusting the tubing length. Residence times from 0.6 min (*V* = 5 mL) to 11.3 min (*L* = 90 mL). With increasing residence time, particle core size increased. Off optimal process window, size control was limited, and the particle homogeneity decreased (**a** and **e**); **f**: Resulting mean diameter (symbol) and standard deviation (plotted as uncertainty bar) determined by the analysis of *N* > 5000 individual nanoparticles imaged by TEM as a function of residence time *t*_r_. Note, the TEM image analysis of particles synthesized at the lowest residence time *t*_r_ = 0.6 min did not yield reliable core diameter values.

**Figure 8:**
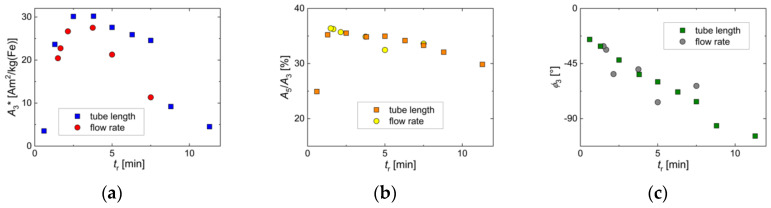
MPS parameters of MNP samples were obtained by continuous synthesis at different residence times *t*_r_ by adjusting the tubing length. For comparison, the corresponding MPS parameters obtained by the flow rate in Section 3.2 were added to the graphs. (**a**) specific amplitude *A*_3_*; (**b**) ratio *A*_5_/*A*_3_; (**c**) phase *ϕ*_3_.

Interestingly, also, at very short residence times below 1 min (*t*_r_ = 0.6 min), MNPs with larger hydrodynamic sizes and broad size distribution were observed in DCS analysis. However, in this case, the agglomeration or aggregation did not seem to be caused by strong magnetic interaction between large magnetic cores but indicated the presence of insoluble intermediate states. The corresponding TEM analysis (Figure 7a) showed aggregates of inhomogeneous particles of different size and morphology, which supported this hypothesis. It has been reported that the reaction mechanism of metal oxide synthesis in aqueous media generally involves hydrolysis of metal salts, followed by dehydration of the resulting hydroxides. A large number of different redox reactions are possible; thus, also hydroxide networks can be formed during hydrolysis [64]. If the residence time was below a critical value, the particle formation process was interrupted at a non-final stage; thus, particles were resulting, which were neither uniform nor electrostatically stabilized. Furthermore, these particles might contain fractions of non-magnetic iron hydroxides, which were able to crosslink and lead to agglomerates (See also Figure 8).

As depicted in Figure 7, the TEM data confirmed the observations of DCS analysis. Increasing *t*_r_ resulted in MNP with larger core sizes. Particles in the optimal process window were relatively homogenous in size and shape. Even the larger particles obtained at *t*_r_ = 11.3 min were still relatively homogenous in size, though shape transition from spherical to more cubic morphology was visible. The core sizes between 13 +/− 3 and 46 +/−7 nm were obtained. The sample synthesized at *t*_r_ = 0.6 min showed inhomogeneous MNP, indicating non-stable intermediate states, as discussed above.

Considering the effect of the residence time variation adjusted by the tubing length on the dynamic magnetic properties of the MNP, we saw a similar behavior as found for *t*_r_ variation by flow rate adjustment in Section 3.2. The three MPS parameters—*A*_3_*, *A*_5_/*A*_3_, and *ϕ*_3_—are shown in Figure 8, together with the results of Section 3.2 (Figure 5). Again, the specific amplitude exhibited a distinct maximum at a residence time *t*_r_ of about 3 min, but with a slightly increased value of *A*_3_* ≈ 30 Am^2^/kg(Fe). The corresponding *A*_5_/*A*_3_ parameter showed the same weak decrease with increasing *t*_r_. At the *A*_3_* maximum, the same *A*_5_/*A*_3_ value of 35% was determined for *t*_r_ adjustment by tubing length as well as by flow rate. The phase *ϕ*_3_ showed a linear (negative) increase with increasing *t*_r_ with a value of about −45° at the *A*_3_* maximum, where *t*_r_ = 3 min. Thus, also, here, we found the core size of about 25 nm (see Figure 7f) and hydrodynamic diameter of about 27 nm (Figure 6b) to be optimum. Comparing the two ways of *t*_r_ selection, the tubing length adjustment seemed to result in a more stable MNP synthesis than by controlling the flow rate, leading to slightly higher MPS values.

The crystallographic analysis by X-ray diffraction (see Figure A3 in the Appendix A) according to the *JCPDS* card no. #*75*-0033 (Fe_3_O_4_) confirmed a pure magnetic phase for three representative samples with core sizes between 25 and 35 nm. All diffraction patterns exhibited the characteristic XRD spectrum of Fe_3_O_4_ (or *γ*-Fe_2_O_3_) nanoparticles without any significant fraction of FeO (indicated by the lack of a (200) peak or *α*-Fe_2_O_3_ (no (104) and (110) peaks) phases [65]. The *γ*-Fe_2_O_3_ phase would exhibit a small peak shift to higher angles and the appearance of two weak characteristic superlattice diffractions from the (210) and (213) planes at lower angles (around 24.8 and 26.8°, respectively). Consistent with this, the samples in this size range obtained by micromixer synthesis exhibited a very high room temperature saturation magnetization in the range 111–117 Am^2^/kg(Fe), as recently published [66]. These values were close to the value for bulk magnetite or maghemite, indicating the high crystallinity of the magnetic phases with a low amount of disorder reached by our synthesis.

### 3.4. Influence of Reaction Temperature T_s_

The reaction temperature *T*_s_ is the second important parameter to control the micromixer synthesis. During the aqueous synthesis of iron oxide nanoparticles, the reaction temperature determines the reaction velocity, particularly during particle growth, since the nucleation is a very fast process even at low temperatures. Therefore, the impact of different reaction temperatures *T*_s_ in the range from 30 °C to 65 °C on particle growth at a fixed residence time of *t*_R_= 3.4 min was investigated. All *T*_s_ were far below the boiling point of water and well above the room temperature (to suppress interfering seasonal variation of *T*_s_ if a sufficient temperature-controlled lab environment could not be maintained). Temperature control in the micromixer set-up was provided by a thermostat bath (Huber), preheated educt solutions, and thin-walled tubing (Teflon material); thus, temperature stability better than 1.5 K was achieved over the whole *T*_s_ range.

**Figure 9:**
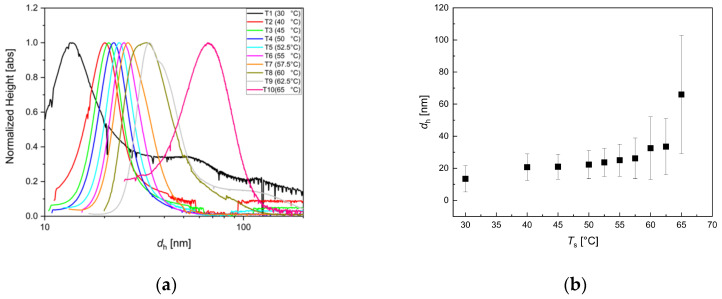
DCS analysis for iron oxide single-core nanoparticles manufactured by continuous micromixer synthesis at *Q* = 8 mL/min total flow rate and *t*_r_ = 3.4 min for different synthesis temperatures *T*_s_ = 30, 40, 45, 50, 52.5, 55, 57.5, 60, 62.5 and 65 °C. (**a**) Hydrodynamic size distribution extracted from DCS measurements; (**b**) mean hydrodynamic diameter *d*_h_ (symbols) and FWHM values (depicted as uncertainty bars) as a function of *T*_s_.

We observed a general trend to yield larger particles at higher *T*_s_, as also reported in the literature for iron oxide particle growth during a hydrothermal process [67]. Higher *T*_s_ enhanced the mobility of active educts and accelerated the particle growth; thus, at an identical residence time, particles grew larger, as long as enough educt material was provided. As displayed in Figure 9, DSC resolved a single fraction of particles with hydrodynamic diameters (peak position) ranging from 12 nm for *T*_s_ = 30 °C up to about 36 nm for *T*_s_ = 62.5 °C with narrow size distribution. Especially in the range from *T*_s_ = 40 °C to 57.5 °C, the hydrodynamic size smoothly increased, exhibiting a narrow and nearly constant size distribution. Particles synthesized at higher temperatures showed a tendency for the formation of suprastructures, e.g., chains of several particles, visible in the DCS data by peak broadening and a shoulder at higher diameters.

For the highest reaction temperature, *T*_s_ = 65°C, MNP with hydrodynamic diameters even about 70 nm and a much broader distribution were detected. This indicated the growing tendency of aggregation due to the increasing magnetic particle-particle interaction of MNP of this size, which no longer could be prevented with the chosen shell stabilization (surfactant layer) [68]. For the lowest reaction temperature *T*_s_ = 30 °C, the formation of aggregates was clearly visible by a distinct second broad distribution with a maximum at about 50 nm. Here, a similar effect, as at very low residence times discussed above, could cause this aggregation behavior. If the reaction temperature was too low at a given residence time, the dynamic particle formation process might not be finalized within this time frame, resulting in intermediate growth states with an inhomogeneous structure, which were insufficiently stabilized during particle growth. Thus, the subsequent addition of a protective layer could not avoid agglomeration efficiently.

**Figure 10:**
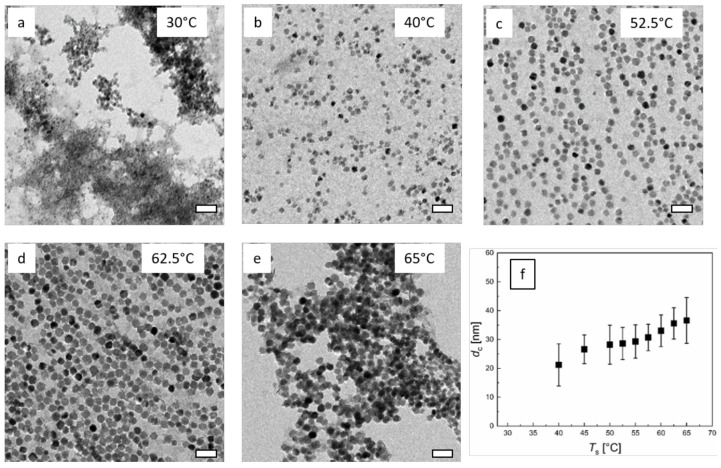
TEM images of continuous micromixer-synthesized nanoparticles at *Q* = 8 mL/min total flow rate and *t*_r_ = 3.4 min. **a**–**e**: Variation of synthesis temperature *T*_s_. Images of MNP obtained by continuous synthesis at defined reaction temperatures in the range of 30 °C to 65 °C. The scale bar is 100 nm. **f**: Resulting mean diameter (symbols) and standard deviation (uncertainty bars) determined by the analysis of *N* > 5000 individual nanoparticles imaged by TEM as a function of *T*_s_.

The TEM images confirmed the results obtained by DCS measurements. The core size of the monotonic increased with increasing *T*_s_. The TEM analysis, shown in Figure 10f, revealed mean cores sizes from less than 20 nm to almost 40 nm for the *T*_s_ range. For particles synthesized at *T*_s_ = 30 °C, TEM clearly showed that at this temperature, the residence time of *t*_r_ = 3.4 min was too short for obtaining homogeneous well-separated single-core nanoparticles but resulted in a significant amount of agglomerated small size iron oxide structures with rather diffuse morphology (Figure 10a). Therefore, a large proportion of the sample could not be considered as isolated single nanoparticles. Besides, the geometry of MNP synthesized at *T*_s_ =30 °C was not very uniform, containing spherical as well as disk-like particle shapes. Furthermore, the particle concentration obtained after magnetic separation was very low, which indicated the presence of a significant amount of poor-quality magnetic particles.

The mean core diameter obtained by TEM image analysis of N > 5000 single MNP ranged from 21.2 nm for *T*_s_ = 40 °C up to 36.6 nm *T*_s_ = 65 °C, smoothly increasing with increasing *T*_s_, as shown in Figure 10f. The standard deviation (displayed as uncertainty bars in Figure 10f) remained quite constant at about 5.5 nm in the central range of *T*_s_ = 40 °C to 62.5 °C. Only for syntheses at the lowest and the highest reaction temperature, the standard deviations in the core size analysis increased to 7.3 nm for *T*_s_ = 30 °C and 7.9 nm for *T*_s_= 65 °C, respectively. The corresponding relative standard deviations (*σ* = mean/standard deviation) of about 0.25 were determined. The number of particles per unit TEM grid area clearly increased with increasing *T*_s_, as can be seen in Figure 10. Regarding the core sizes accessible by the micromixer synthesis of up to about 40 nm, we assumed a single domain magnetic structure of the single-core iron oxide MNP. Theoretically, a critical core size for forming a multi-domain structure larger than 75 nm for cubic and even larger than 120 nm for spherical particle shape has been estimated [69]. Whereas experimentally, a much lower border between single and multidomain structure at critical sizes in the range 30 nm to 50 nm has been reported for cubic MNP. Therefore, a fraction of particles with a multi-domain structure cannot be excluded, which would require further analysis methods to clarify.

From DCS and TEM measurements, we identified an optimal process operation window between *T*_s_ = 40 °C and 60 °C, in which tunable MNP with core sizes between 20 nm and 35 nm could be produced that were stable in an aqueous suspension medium. Particles of this size range are particularly interesting for versatile biomedical applications like magnetic fluid hyperthermia, magnetic separation, or drug targeting, where huge magnetic moments are advantageous [70]. In a recently published paper, we investigated single-core magnetic nanoparticles of this size range for their capacity in biomedical applications as MRI contrast agents, as tracer materials for magnetic particle imaging, or as heating agents for hyperthermia applications [66]. For instance, for single-core nanoparticles with an average core diameter of 30 nm, relaxivities of r_1_ = 8.8(1) L mol^−1^ s^−1^ and r_2_ = 289(8) L mol^−1^ s^−1^ were determined. This clearly exceeded the r_2_ value of Resovist^®^ (r_2_ = 95 L mol^−1^ s^−1^, reported in the literature at the same field B_0_ = 1.5 T) [71], indicating a good performance as a negative contrast agent. Usually, single-core iron oxide nanoparticles in this size range are not easily accessible by other synthesis methods, especially nanoparticles that are stably dispersed in an aqueous environment. For instance, Hufschmid et al. presented a method based on thermal decomposition envisaging this specific core size range. Producing a set of particles in this size range required numerous synthesis runs, each with different educt solutions and reaction times for more than 10 h, followed by time-consuming phase transfer to aqueous media [72].

**Figure 11:**
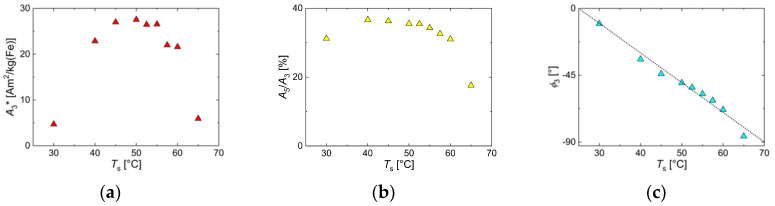
MPS parameters of MNP samples obtained by continuous synthesis at different synthesis temperatures *T*_s_. (**a**) Specific amplitude *A*_3_*; (**b**) ratio *A*_5_/*A*_3_; (**c**) phase *ϕ*_3_. The dotted line in (**c**) displays the phenomenological linear relation (*ϕ*_3_ = 50° − 2·*T*_s_) between phase and *T*_s_.

To assess the magnetic behavior that can be adjusted by *T*_s_ variation, we carried out MPS and ACS measurements. The dynamic magnetic response at *B*_e_ = 25 mT, parametrized by the three characteristic MPS quantities—the specific amplitude *A*_3_*, the ratio *A*_5_/*A*_3_, and the phase *ϕ*_3_ as a function of *T*_s_—is summarized in Figure 11. The nanoparticles synthesized at *T*_s_ = 30 °C exhibited a rater low *A*_3_* value of about 5 Am^2^/kg(Fe), indicating a low magnetic moment of the MNP responding to the dynamic excitation at *f*_0_ = 25 kHz. For *T*_s_ = 40 °C and higher, *A*_3_* significantly increased far above 20 Am^2^/kg(Fe) due to the increasing size and more uniform morphology compared to particles synthesized at *T*_s_ = 30 °C. The magnetic response reached a maximum at about *T*_s_ = 50 °C, and for higher temperatures, *A*_3_* gradually decreased again. Though the particle moments still increased in size, they became too slow to follow the excitation frequency. At the highest temperature of *T*_s_ = 65 °C, *A*_3_* had dropped down to a value slightly above 5 Am^2^/kg(Fe). The (concentration-independent) harmonic amplitude *A*_5_/*A*_3_ showed the same behavior as a function of *T*_s_ with a maximum value of about 38% at *T*_s_ = 42 °C, but with less pronounced reduction at the lowest and highest temperatures. The phase angle *ϕ*_3_ showed a linear (negative) increase with increasing *T*_s_ passing −45° at the maximum values of *A*_3_ and *A*_5_/*A*_3_, indicating that for angles larger than −45°, the moments could not follow the excitation fast enough and were thereby reducing *A*_3_* and *A*_5_/*A*_3_.

**Figure 12:**
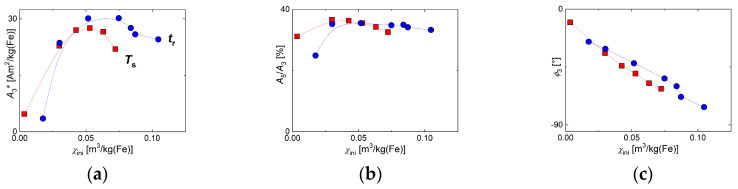
MPS parameters of MNP samples obtained by continuous synthesis at different reaction temperatures *T*_s_ (red squares) and residence time *t*_r_ adjusted by tubing length (blue circles) plotted against the corresponding initial susceptibility *χ*_ini_ determined by alternative current susceptibility (ACS). (**a**) Specific amplitude *A*_3_*; (**b**) ratio *A*_5_/*A*_3_; (**c**) phase *ϕ*_3_. Note the lines are guide to the eyes.

MPS and ACS results could be combined by relating them to the corresponding residence times *t*_r_ or synthesis temperatures *T*_s_, respectively. Hence, the MPS parameters—*A*_3_*, *A*_5_/*A*_3_, and *ϕ*_3_ as a function of the initial susceptibility *χ*_ini_ obtained by ACS (Appendix A Figure A2)—are displayed in Figure 12 for the different micromixer synthesis parameters. The initial susceptibility *χ*_ini_, reflecting the moment of the MNP achieved at a synthesis parameter *t*_r_ or *T*_s_, served as the x-axis for the three MPS parameters—*A*_3_*, *A*_5_/*A*_3_, and *ϕ*_3_. It showed that synthesis temperature *T*_s_ and residence time *t*_r_ impacted the magnetic properties in a slightly different manner. Though the curves for both parameters showed a very similar shape, the maximum in *A*_3_* was reached at lower *χ*_ini_ values. The same was observed for the *A*_5_/*A*_3_ parameter.

The results indicated the significant influence of *T*_s_ on the reaction, the obtained single-core nanoparticle structures, and finally, the resulting magnetic characteristics determined by size, size distribution, stability, and magnetic properties. Increasing the reaction temperature of the micromixer synthesis already in a very narrow interval of 2.5 K significantly affected the MNP characteristics and confirmed the rapid and efficient heat transfer of the continuous micromixer synthesis. Compared with this, the residence time *t*_r_ seemed to have a finer setting of magnetic parameters with slightly higher *A*_3_* values than obtained by *T*_s_ adjustment. Furthermore, MPS and ACS were two highly sensitive magnetic techniques capable of resolving the changes in magnetic parameters addressed by *T*_s_ variation.

### 3.5. Online-MPS as a Valuable Tool for Efficient Magnetic Process Analysis During the Continuous, Tunable Synthesis of High-Quality Single-Core MNP by Micromixer Synthesis

Though MPS is a powerful technique to evaluate the dynamic magnetic behavior of the micromixer synthesis products, it generally requires taking individual samples after each synthesis. Nevertheless, the short measurement time of a few milliseconds combined with the extremely high sensitivity to detect magnetic moments below 10^−11^ Am^2^ makes the MPS technique ideally suited to detect the magnetic properties during the entire micromixer synthesis process. For this purpose, a compact MPS device was developed capable of easy looping into the dwell-zone tubing system used to adjust the residence time during micromixer synthesis. The setup and operation of this novel device are described in detail in the following article of this volume [61]. For the first demonstration of the capability of this device for magnetic process control, we performed a micromixer synthesis run, where *T*_s_ was stepwise increased by 5 K (except for an intermediate 2.5 K temperature step at 52.5 °C) every 10 min starting at *T*_s_ = 30 °C while keeping all other synthesis parameters constant. During the complete synthesis run, the online-MPS device was looped into the growth stage tubing used for residence time adjustment, and MPS measurements were continuously acquired with a temporal resolution of Δ*t* = 1 s at an excitation field strength of *B*_e_ = 12 mT.

**Figure 13:**
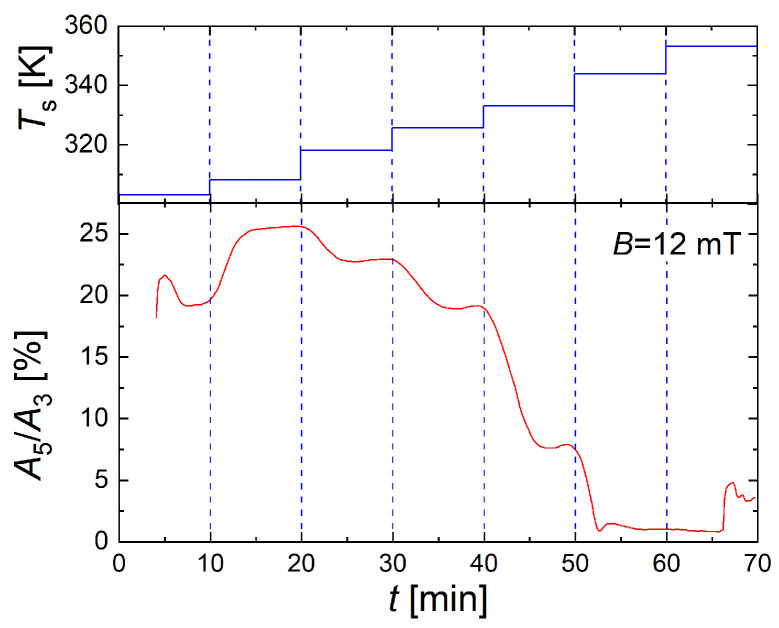
Monitoring of micromixer synthesis by online-MPS. The concentration-independent MPS parameter *A*_5_/*A*_3_ directly reflected changes in the magnetic properties during MNP synthesis, here provoked by step-wisely increasing *T*_s_. Every 10 min, *T*_s_ (top panel, blue line) was increased by 5 K (with one additional intermediate *T*_s_ setpoint at 52.5 °C) starting at *T*_s_ = 30 °C. The synthesis started at *t* = 0 and was monitored by continuous MPS acquisition (Δt = 1 s, *B*_e_ = 12 mT) to obtain the parameter *A*_5_/*A*_3_ values (bottom panel, red line). The synthesis was performed at *Q* = 8 mL/min total flow rate and *t*_r_ = 3.4 min.

Figure 13 displays the MPS parameter *A*_5_/*A*_3_ (red line) during the synthesis run, sensitively reflecting changes in the magnetic properties directly after each increase of *T*_s_ (red line). Starting the synthesis at *t* = 0, it took about 4 min (at 30 °C) until the first reliable MPS spectra with *A*_5_/*A*_3_ values above noise floor appeared, which, then after a small overshoot within the following 4 min approach, reached a stable value of about 20%. With the next *T*_s_ set-point at 35 °C, a significant increase in *A*_5_/*A*_3_—maximum value of about 25%—was observed, while for all higher *T*_s_ values, *A*_5_/*A*_3_ dropped again. Roughly, this behavior was in accordance with the MPS results of the previous Section 3.3. Note, the maximum value detected inside the dwell-zone tubing by online-MPS was significantly lower than the maximum found by MPS of extracted individual samples of about 35%. The reason is that for the online MPS measurements, we chose a much lower excitation field amplitude (*B*_e_ = 12 mT) than for all other MPS measurements (*B*_e_ = 25 mT), which mainly explains the difference in the absolute values of *A*_5_/*A*_3_. By this excitation field reduction, we intended to avoid any additional heat transfer from the excitation coil of the online-MPS device. Additional experiments are envisaged to further characterize any heat transfer effects of the coil at higher temperatures on micromixer synthesis in the growth stage tubing.

The general benefit of probing the magnetic properties inline during synthesis has been demonstrated in [73], where an NMR detection scheme was developed and tested for online characterization of iron oxide nanoparticles synthesized in a flow-based microreactor. To this end, an automated NMR relaxometer device was presented to determine the transversal and longitudinal relaxation times of magnetic iron oxide nanoparticles obtained by online flow-based microreactor synthesis. Though this system showed very high sensitivity, it requires a rather long time (minutes) to determine the relaxation times compared to the MPS measurement time of the system presented here. Furthermore, the magnetic field of 0.5 T required to detect the proton resonance was much higher than the excitation field of some tens of millitesla and might lead to aggregation or chain formation of the MNP during the NMR-measurements. However, both techniques are efficient tools capable of speeding up synthesis optimization and product characterization of MNP to be developed for biomedical, theranostic, and biosensing applications.

## 4. Summary and Conclusions

In this work, the continuous micromixer synthesis of single-core iron oxide magnetic nanoparticles was presented and compared with batch synthesis. Continuous micromixer synthesis has many advantages over conventional batch synthesis regarding reproducibility, simplicity, time exposure, quality of the product, and upscaling capability of the procedure. The influence of the two (most) important synthesis parameters—the reaction temperature and the residence time (either adjusted by the total flow rate or the tubing volume)—of the reaction was studied. The impact of *T*_s_ and *t*_r_ on size, size distribution, and the stability of the MNP was directly reflected in the magnetic properties measured by magnetic particles spectroscopy and AC-susceptibility measurements. In this way, the tunability of our approach was clearly demonstrated since, by variation of input parameters, the resulting product properties are changed in a unique and highly reproducible way.

The operation of the newly developed benchtop MPS device connected to the growth stage of the micromixer platform directly probing the magnetic properties during the synthesis pointed out the great potential of this highly sensitive and fast magnetic technique. Once established as an integrated inline analytic process tool, it could substantially accelerate particle development while monitoring the magnetic quality of the nanoparticles.

The advantage of the micromixer technology is that the influence of different process parameters can be investigated comprehensively to gain a better process understanding. With this, the superiority of microfluidics can be fully exploited, i.e., its excellent mass and heat transfer and its rapid and controlled synthesis conditions. We understand that our micromixer approach in this way is unique, that is, it is tunable: by variation of input parameters, the resulting product properties are manipulated in a highly reproducible way. Moreover, the micromixer technology is capable of operating under nearly identical process conditions at large scales (at least in the decaliter range), as well.

The presented continuous micromixer technology to produce high-quality single-core iron oxide magnetic nanoparticles might become a valuable tool to promote the standardized manufacturing of magnetic nanoparticle systems for nanomedicine applications, thereby fostering the translation of laboratory nanoparticle systems into clinical products, including safety, regulatory, and ethical requirements.

## Appendix A

The magnetic AC susceptibility (ACS) was a complex signal measured in the form *χ* = *χ*′ + i*χ*″, where the real part *χ*′ denoted the susceptibility component of the nanoparticle moments that was in-phase with the applied AC magnetic field, and the imaginary part *χ*″ was the corresponding out-of-phase component. The relative ratio of the two components revealed the phase lag between particle moment and applied AC field, and this led for *χ*′ = *χ*″ to a 45° phase angle. The maximum of *χ*″ was reached for blocked nanoparticles (no Néel relaxation, e.g., iron oxide cores with diameters larger 20 nm at room temperature) when the Brownian relaxation time was equal to the rotational cycle time of the applied field. This was revealed as a peak in the *χ*″ susceptibility component as a function of frequency. For larger hydrodynamic sizes, e.g., for longer Brownian relaxation times, this peak shifted to lower frequencies. Thus, ACS measurements as a function of frequency enabled the assessment of hydrodynamic sizes of magnetic nanoparticles.

Figure A1 shows the ACS signals normalized to the iron amount of single-core iron oxide nanoparticles synthesized by continuous micromixer synthesized at different residence times *t*_r_ (adjusted by tube length) as a function of frequency. At low frequencies, *χ*′ was constant, e.g., the moments could follow the AC field, from which the initial susceptibility *χ*_ini_ could be determined, extrapolating *χ*′ for *f*→0. At higher frequencies, *χ*′ was gradually decreasing, and the larger moments no longer could follow the excitation, leading to a phase lag, and thus the imaginary part *χ*″ was increasing, showing a pronounced peak at the inflection point of *χ*′. With increasing residence time *t*_r_, e.g., increasing core particle size, the initial susceptibility *χ*_ini_ increased. At the same time, the peak in *χ*″ was shifted towards lower frequencies because with increasing core size, the hydrodynamic diameter increased, as well.

Similar behavior was observed for the ACS signals normalized to the iron amount of single-core iron oxide nanoparticles synthesized by continuous micromixer at different reaction temperatures *T*_s_. As displayed in Figure A2, here again, an increase of *χ*_ini_ with increasing *T*_s_ was observed. But for *T*_s_ ≥ 60 °C, *χ*_ini_ dropped again. The sample synthesized at *T*_s_ = 65 °C showed an aggregation tendency since it exhibited a non-zero *χ*″ component and no constant plateau in the *χ*′ component for lower frequencies.

The initial susceptibility *χ*_ini_ was proportional to the (mean) size of the magnetic nanoparticle moments and could be related to the characteristic three MPS parameters—*A*_3_*, *A*_5_/*A*_3_, and *ϕ*_3_—via the corresponding residence time *t*_r_ or synthesis temperature *T*_s_, as shown in Figure 12. This allowed the magnetic assessment of the impact of *T*_s_ and *t*_r_ variation on the magnetic behavior of the resulting nanoparticles.

To assess the crystal structure and iron oxide phase of samples manufactured at three different core diameter sizes in the range 25 nm to 35 nm by variation of the residence time, XRD 2*θ* wide-angle scans were carried out, as shown in Figure A3. The corresponding indices of a magnetite crystal structure (which are nearly the same as for *γ*-Fe_2_O_3_) at the main peak positions were taken from the *JCPDS* card no. #*75*-0033 (Fe_3_O_4_) and # 39-1346 (*γ*-Fe_2_O_3_).
